# New Insight into Ki67 Expression at the Invasive Front in Breast Cancer

**DOI:** 10.1371/journal.pone.0054912

**Published:** 2013-01-31

**Authors:** Peng Gong, Yingxin Wang, Gavin Liu, Jing Zhang, Zhongyu Wang

**Affiliations:** Hepatobiliary Surgery, the First Affiliated Hospital of Dalian Medical University, Dalian, Liaoning, China; University of North Carolina School of Medicine, United States of America

## Abstract

**Purpose:**

To investigate the distribution of Ki67+ cells in breast cancer in relation to clinical-pathological parameters and prognosis.

**Materials and Methods:**

Ki67 expression status was detected in 1,086 breast cancer specimens using immunohistochemistry staining and examining the relationship between the Ki67+ cells' location. Subsequently, clinical-pathological parameters and prognosis were determined.

**Results:**

In total, Ki67 protein expression was found in 781 (71.92%) of the 1,086 breast cancer specimens. Among the 781 Ki67+ cases, 461 were defined as diffuse type and 320 were defined as borderline type. After universal correlation analysis, significant differences were observed in age, histological grade, metastatic nodes, postoperative distant metastasis, and molecular subtype between Ki67+ and Ki67− cases (*P* = 0.01, 0.001, 0.001, 0.001, and 0.001, respectively). After subgroup analysis, the borderline cases were found to be characterized by a high distant metastasis rate compared to the diffuse cases as well as the Ki67− cases (*P* = 0.001). No differences were observed between diffuse type or Ki67− cases (*P* = 0.105). Multivariate analysis showed that age, tumor size, histological grade, lymph node metastasis, molecular subtype, and the Ki67 distribution pattern were observed to be related to postoperative distant metastasis (all *P*<0.05). Furthermore, borderline type was shown to attain a significantly more distant bone and liver metastasis and worse disease-specific survival than the other types (*P* = 0.001). In the Cox regression test, the Ki67 distribution pattern was detected as an independent prognostic factor (*P* = 0.001).

**Conclusion:**

The distribution pattern of Ki67 may be a new independent prognostic factor for breast cancer.

## Introduction

Breast cancer is one of the most common cancers and the leading cause of cancer-related death in women [1], [2]. In 2008, 1,380,000 new occurrences of breast cancer were diagnosed worldwide, with 458,400 persons dying from breast cancer that same year [3], [4]. Death usually results from uncontrolled metastatic disease rather than local recurrence [5]. The most common organ metastases in patients with breast cancer are bone, lung, and liver [6]. Although the 5-year survival rate for patients with breast cancer approaches 80%, in metastatic patients, it decreases to 20% [7]. Thus, detecting distant metastasis early is especially important to improve the breast cancer patient's prognosis.

Several studies have investigated the markers that predict distant metastasis in breast cancer. Ki-67 is a protein that in humans is encoded by the MKI67 gene [8]. Antigen Ki-67 is a nucleus protein that is strictly associated with and may be necessary for cellular proliferation [9]. It was recently reported that Ki67 might be suitable for including in the routine clinical practice of breast cancer. In novel multigene tests, however, proliferation has a major impact on calculating the risk of recurrence [10]. For example, Ki67 independently improved the prediction of treatment response and prognosis in a group of breast cancer patients receiving neoadjuvant treatment [11].

Currently, the expression location status and the clinical implications of Ki67 in breast cancer have never been studied. In preliminary experiments, we investigated that Ki67 expression was distributed in different ways in different cases. Hence, we extend our studies to include the relationship between the distribution type of Ki67 expression and biological behaviors and prognosis in breast cancer. Gaining this knowledge will lay a foundation for managing breast cancer.

## Methods

### Patients and tissue specimens

A total of 1,086 patients who had histologically confirmed breast cancer and who underwent radical operations in the Tumor Hospital of Liaoning province and China Medical University between January 2001 and January 2006 were enrolled for immunohistochemical and immunofluorescence double staining and prognostic analysis. The mean age of patients was 50.73±10.28 years (range from 27 to 80 years). Of the 1,086 patients, 544 were with Luminal A tumors and 135 with Luminal B tumors, 102 were with HER2-enriched type tumors, and 305 were with basal-like type tumors [12]. The criteria to include a patient in the present study were as follows: (1) curative operations were performed; (2) resected specimens were pathologically examined; (3) more than 10 lymph nodes were pathologically examined after the operation; and (4) a complete medical record including the status of ER, PR, Her2, p53, and Ki67 was available. The study protocol was approved by the Ethics Committee of Dalian Medical University and Liaoning Tumor Hospital, and written informed consent was obtained from all participants. The study was also approved by the Ethics Committee of China Medical University.

### Immunohistochemistry experimental procedures

We fixed thin slices of tumor tissue from all cases received in our histopathology unit in 4% formaldehyde solution (pH 7.0) for periods not exceeding 24 h. The tissues were processed routinely for paraffin embedding, and 4 μm-thick sections were cut and placed on glass slides coated with 3-aminopropyl triethoxysilane for immunohistochemistry. Tissue samples were stained with hematoxylin and eosin to determine histological type and grade of tumors.

Breast tumor tissues were cut at a thickness of 4 μm using a cryostat. The sections were mounted on microscope slides, air dried, and then fixed in a mixture of 50% acetone and 50% methanol. The sections were then de-waxed with xylene, gradually hydrated with gradient alcohol, and washed with PBS. Sections were incubated for 60 min with the Ki67 antibody (Santa Cruz Biotechnology, Inc. USA; sc-15402). Following washing with PBS, sections were incubated for 30 min in the secondary biotinylated antibody (rabbit anti-mouse IgA-B, Santa Cruz Biotechnology, Inc. USA; sc-358961). Following washings, Avidin Biotin Complex (1:1,000 dilution) was then applied to the sections for 30 to 60 min at room temperature. The immunoreactive products were visualized by catalysis of 3, 3-diaminobenzidine (DAB) by horseradish peroxidase in the presence of H_2_O_2_ following extensive washings. Sections were then counterstained in Gill's hematoxylin and dehydrated in ascending grades of methanol before clearing in xylene and mounting under a coverslip.

Ki67 expression was classified semi-quantitatively according to the following criteria: We considered samples as Ki67 positive if more than 1% of neoplastic cells discretely expressed Ki67 in their nucleus. The invasive front of the tumor was defined as the three- to six layers of tumor cells at the front edge or the scattered tumor groups between the tumor and the host tissue or organ. Firstly, two pathology experts were invited to assess for distribution of positive cells into borderline type and diffuse type. The third pathology expert will be invited to re-evaluate the distribution type if they got different result. The Ki67 positive cases with Ki67+ tumor cells found in the invasive front tumor area were two times more prevalent than the Ki67+ tumor cells in the non-invasive front tumor area were defined as borderline type. In contrast, the Ki67 positive cases with the Ki67+ tumor cells found in the invasive front tumor area were not two times more prevalent than the Ki67+ tumor cells in the non-invasive front tumor area were defined as diffuse type [13].

### Statistical Analysis

All data were analyzed with SPSS Statistics software (Version 13.0, Chicago, IL, USA). Relationships between Ki67 and other parameters were studied using the chi-square test, Fisher's extract test, or independent *t* tests. Disease-specific survival was analyzed using the Kaplan-Meier method. The log-rank test was used to analyze survival differences. Multivariate analysis was performed using the Cox proportional hazards model selected in forward stepwise. A *P* value of less than 0.05 was considered statistically significant.

## Results

### Ki67 expression in breast cancer and the relationship between Ki67 and clinicopathological characteristics

**Figure 1:**
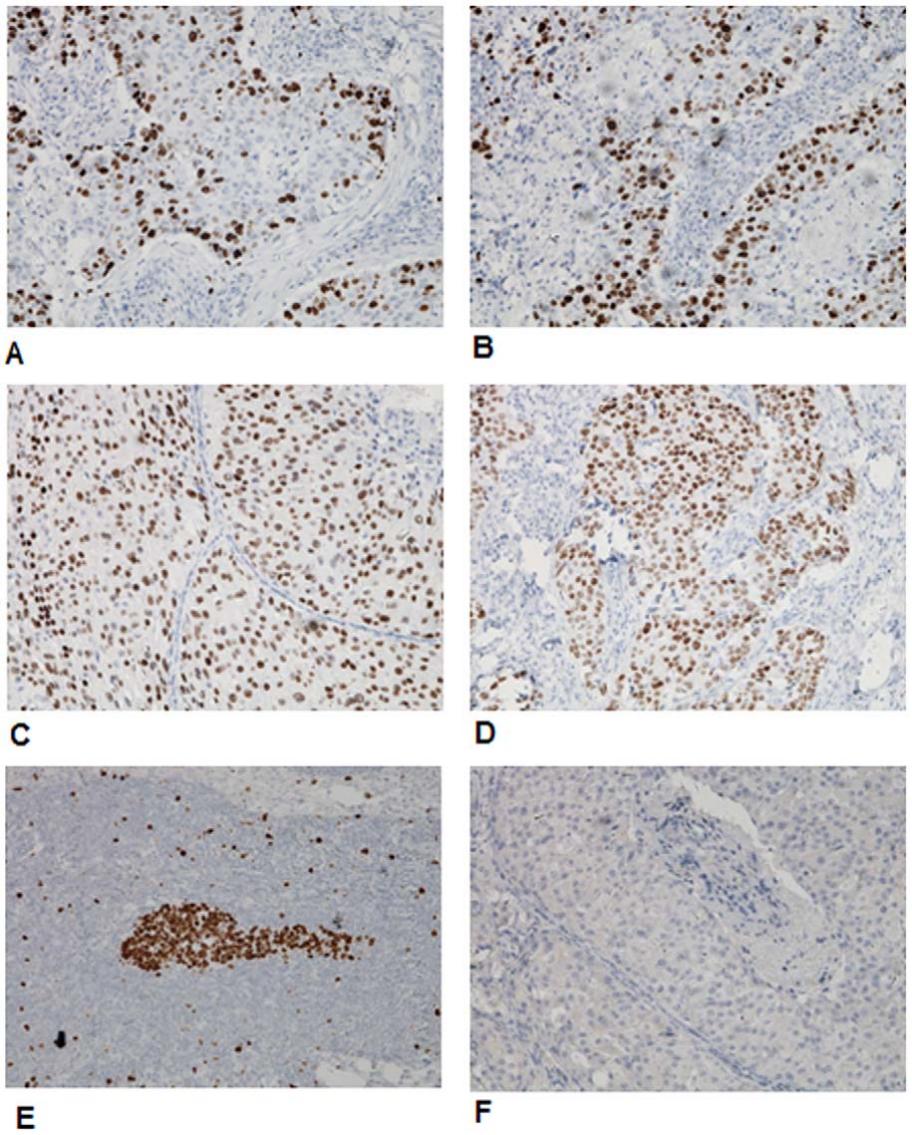
Ki67 protein was located at nucleus of the breast cancers. A(2+) and B(2+), Ki67 protein distributed as diffuse type; C(2+) and D(2+), Ki67 protein distributed as borderline type; E(2+), Ki67 protein expressed in the lymph node metastasis; F, negative control.

The mean age of the 1,086 patients studied was 50.73 years (range: 27–80 years). Within the total sample, 519 (47.79%) patients had lymph node metastasis and 288 (26.52%) exhibited postoperative distant metastasis (Table 1). In total, Ki67 protein expression was found in 781 (71.92%) of the 1,086 breast cancer specimens. Among the 781 Ki67 positive cases, 461 cases were defined as diffuse type and 320 cases were defined as borderline type (Figure 1). After universal correlation analysis, significant differences were observed in age, histological grade, metastatic nodes, postoperative distant metastasis, and molecular subtypes between Ki67+ and Ki67− cases (*P* = 0.01, 0.001, 0.001, 0.001 and 0.001, respectively), while these differences were not observed in tumor size (*P* = 0.118) (Table 1). There were significant difference in the Ki67 distribution pattern among age, histological grade, metastatic nodes, postoperative distant metastasis, and molecular subtypes in Ki67+ cases (*P* = 0.001, 0.001, 0.002, 0.001 and 0.001, respectively).

**Table 1:** Correlations between distribution pattern of Ki67 expression and clinic-pathological features (n = 1086).

Variables	n	Ki67−	Ki67^+^		*P_1_* value	*P_2_* value	*P_3_* value
			Diffuse type	Borderline type			
Age					0.01	0.326	0.001
<40 Y	174	35	42	97			
>40 Y	912	270	419	223			
Tumor size					0.433	0.482	0.118
T1	173	56	71	46			
T2	836	229	365	242			
T3	69	19	22	28			
T4	8	1	3	4			
Histological grade					0.001	0.001	0.001
I	84	38	20	26			
II	732	226	374	132			
III	270	41	67	162			
Metastatic nodes					0.001	0.021	0.002
negative	567	188	245	134			
positive	519	117	216	186			
Distant metastasis					0.001	0.105	0.001
negative	798	258	368	172			
positive	288	47	93	148			
Molecular Subtypes					0.001	0.05	0.001
Luminal A	544	176	282	86			
Luminal B	135	42	71	22			
HER2-enriched type	102	25	48	29			
basal-like type	305	62	60	183			

*P_1_*_,_ Ki67^+^ group compared to Ki67^−^ group; *P_2_*_,_ Diffuse type compared to Ki67− group; *P_3_*_,_ borderline type compared to diffuse type.

### The relationship between Ki67 expression type and postoperative distant metastasis

Multivariate analysis showed that age, tumor size, histological grade, lymph node metastasis, molecular subtypes, and Ki67 distribution pattern related to postoperative distant metastasis (*P* = 0.001, 0.035, 0.001, 0.001, 0.001, and 0.001, respectively) (Table 2). After subgroup analysis, borderline type cases showed a high distant metastasis rate compared to diffuse type, as well as Ki67− cases (*P* = 0.001), while no differences were observed between diffuse type or Ki67− cases (*P* = 0.105). Multivariate analysis showed that age, tumor size, histological grade, lymph node metastasis, molecular subtypes and Ki67 distribution pattern was observed to be related to postoperative distant metastasis (all *P*<0.05).

**Table 2:** Multivariate analysis of the factors related to post-operative distant metastasis.

Characteristic	OR	95% CI for OR	*P* value
age	0.335	0.211–0.532	0.001
Tumor size	1.475	1.029–2.114	0.035
Histological grade	3.574	2.540–5.028	0.001
Lymph node metastasis	4.836	3.239–7.219	0.001
Molecular subtypes	7.515	5.263–11.780	0.001
Ki67 distribution pattern	4.662	2.863–6.928	0.001
Constant	0.005		

OR, odd ratio.

We also investigated the postoperative distant metastasis rates among the different groups identified. Cases with positive Ki67 expression exhibited a significantly higher postoperative distant metastasis rate compared to those without Ki67 expression (36.27% vs 19.86%, *P* = 0.01). Furthermore, the borderline type was shown to attain a significantly more distant bone metastasis (39.78% vs 52.70% for diffuse type vs. borderline type) and liver metastasis (13.98% vs 17.39% for diffuse type vs. borderline type) (Table 3).

**Table 3:** Correlations between Ki67 distribution pattern and distant metastasis (n(%)).

Organs metastasis	Diffuse type(93/461)	Borderline Type(148/320)
Bone	37(39.78%)	78(52.70%)
Lung	29(31.18%)	19(12.84)
Liver	13(13.98%)	32(17.39)
Ovarian	4(4.3%)	11(7.43)
Others	7(7.5%)	8(5.41)

### Prognostic analysis

**Figure 2:**
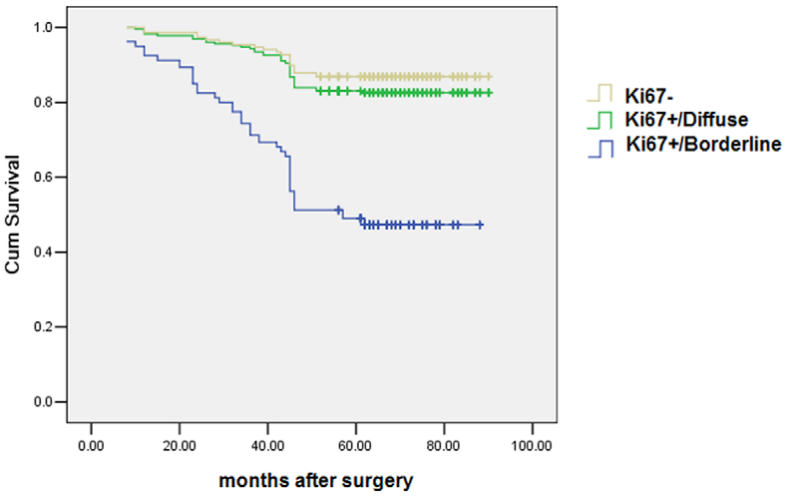
Brderline type cases attain a significantly worse disease-specific survival than the diffuse type or Ki67-cases (*P* = 0.001).

The Kaplan-Meier method for survival analysis showed that borderline type attained a significantly worse disease-specific survival than the other types (*P* = 0.001) (Figure 2). In the Cox regression test, the Ki67 distribution pattern was detected as an independent prognostic factor (*P* = 0.001) (Table 4).

**Table 4:** Cox model regression analysis of the breast cancer prognostic factors.

Varies	OR	95% CI for OR	*P* value
age	1.410	1.133–1.755	0.002
Histological type	1.545	1.355–1.762	0.001
Lymph node metastasis	1.245	1.074–1.443	0.004
Molecular subtypes	2.720	1.907–3.284	0.001
Ki67 distribution pattern	3.178	1.652–5.945	0.001

## Discussion

It has been acknowledged that the Ki67 protein is strictly associated with cell proliferation [14]. During interphase, the antigen can be detected exclusively within the nucleus, whereas in mitosis, most of the protein is relocated to the surface of the chromosomes. The fact that the Ki67 protein is present during all active phases of the cell cycle (G(1), S, G(2), and mitosis), but is absent from resting cells (G(0)), makes it an excellent marker to determine the so-called growth fraction of a given cell population [15]. Moreover, Ki67 is one of the 21 prospectively selected genes of the Oncotype DXTM assay used to predict the risk of recurrence in a node-negative, tamoxifen-treated breast cancer population enrolled in the National Surgical Adjuvant Breast and Bowel Project B-14 (NSABP B-14). It is also used to predict the magnitude of chemotherapy benefit in women with node-negative, estrogen receptor (ER)-positive breast cancer enrolled in the NSABP B-20 trial [16].

It has been suggested that tumor cells might show cellular dedifferentiation in the invasive front area, with loss of an epithelial phenotype and a gain of a mesenchymal phenotype, which facilitates invasive and metastatic growth of originally differentiated cancer cells. The malignant progression is epithelial-mesenchymal transition (EMT) [13]. In the present study, we found that Ki67 expression distributed in a different manner in different cases. In most of the cases, the nucleus positive Ki67 was diffusely located in the tumor, whereas in other cases the nucleus positive Ki67 only expressed in the invasive front of the breast cancer (borderline type). Different biological behaviors may exist for the different types of Ki67 positive cases.

Finally, 71.92% of the enrolled breast cancer samples positively expressed Ki67 protein in the present study. A subset of 461 cases was defined as diffuse type and 320 cases were defined as borderline type. Interestingly, the borderline type cases were found to have a high distant metastasis rate compared to the diffuse type, as well as Ki67− cases. In contrast, no differences were observed between diffuse type or Ki67− cases. Multivariate analysis showed that age, tumor size, histological grade, lymph node metastasis, triple-negative breast cancer, and Ki67 distribution pattern were observed to be related to postoperative distant metastasis. Furthermore, borderline type was shown to attain significantly more cases of distant bone and liver metastasis and worse disease-specific survival than the other types.

In a recent study, Delpech et al. [17] reported that high Ki67 expression in the primary tumor remained an independent, adverse prognostic factor in metastatic disease. Low Ki67 expression in the primary tumor is associated with higher clinical benefit, a longer time to progress to first-line endocrine therapy, and longer survival after metastatic recurrence [17].

After investigating the relationship between nucleus β-catenin expression in the invasive front of colorectal cancers and liver metastatic lesions and other clinicopathological characteristics, Wang et al. [18] observed that over-expression of nucleus β-catenin at the invasive front in colorectal cancer was strongly associated with and may be a promising predictor of liver metastasis. Ki67 is considered to be a protein associated with cell cycle activity and shows a good correlation with the growth fraction and has been proposed as a prognostic or predictive marker in breast cancer. According to our knowledge, however, this is the first study to elucidate the relationship between nucleus Ki67 over-expression distribution type in breast cancer and synchronous liver metastasis based on clinical and pathological data. We demonstrated that over-expression of nucleus Ki67 at the invasive front in breast cancer is associated with bone and liver metastasis. The clinical significance of the present study is that we provide a promising predictor of bone and liver metastasis for those affected by breast cancer.
